# Emerging Role of Tripartite Synaptic Transmission in the Pathomechanism of Autosomal-Dominant Sleep-Related Hypermotor Epilepsy

**DOI:** 10.3390/ijms26199671

**Published:** 2025-10-03

**Authors:** Tomoka Oka, Ruri Okubo, Eishi Motomura, Motohiro Okada

**Affiliations:** Department of Neuropsychiatry, Division of Neuroscience, Graduate School of Medicine, Mie University, Tsu, Mie 514-8507, Japan; tomoka-oka@clin.medic.mie-u.ac.jp (T.O.); okubo-r@med.mie-u.ac.jp (R.O.); motomura@clin.medic.mie-u.ac.jp (E.M.)

**Keywords:** ADSHE, tripartite synaptic transmission, L-glutamate, ACh, hemichannel

## Abstract

Autosomal-dominant sleep-related hypermotor epilepsy (ADSHE) was the first distinct genetic epilepsy proven to be caused by mutation of the *CHRNA4* gene, originally reported in 1994. In the past three decades, pathomechanisms of ADSHE associated with mutant nicotinic acetylcholine receptors (nAChRs) have been explored via various studies, including in vitro experiments and genetic rodent models. However, findings emphasize that functional abnormalities of ADSHE-mutant nAChRs alone cannot generate ictogenesis; rather, development of abnormalities in various other transmission systems induced by ADSHE-mutant nAChRs during the neurodevelopmental process before the ADSHE onset is involved in development of epileptogenesis/ictogenesis. Intra-thalamic GABAergic disinhibition induced by loss-of-function of S284L-mutant nAChRs (S286L-mutant nAChRs in rat ADSHE models) contributes to enhancing propagation of physiological ripple-burst high-frequency oscillation (HFO) and Erk signaling during sleep, leading to enhancement of the trafficking of pannexin1, connexin43, and P2X7 purinergic receptor to the astroglial plasma membrane. The combination of activation of physiological ripple-HFO and upregulation of astroglial hemichannels under the GABAergic disinhibition plays an important role in generation of epileptogenic fast-ripple-HFO during sleep. Therefore, loss-of-function of the S284L-mutation alone cannot drive ictogenesis but contributes to the development of epileptogenesis as an initial abnormality. Based on these recent findings using genetic rat ADSHE models, harboring the rat S286L-mutant *Chrna4* corresponding to the human S284L-mutant *CHRNA4*, this report proposes hypothetical pathomechanisms of ADSHE.

## 1. Introduction

Autosomal-dominant sleep-related hypermotor epilepsy (ADSHE), which was previously known as autosomal-dominant nocturnal frontal lobe epilepsy (ADNFLE), is a subfamily of sleep-related hypermotor epilepsy syndrome (SHE) [1,2,3,4]. ADSHE was the first genetic epilepsy identified as being caused by gene mutations in 1994 [2,4,5,6]. Initially, mutations were identified in *CHRNA4* [2,4,5,6,7,8,9,10,11,12,13,14,15,16,17] and *CHRNB2* [18,19,20,21,22,23,24,25], which encode the α4 and β2 subunits of nicotinic acetylcholine (ACh) receptors (nAChRs), respectively. Based on these clinical findings, the majority of epileptologists understood that ADSHE was a channelopathy caused by dysfunction of mutant nAChRs [26,27,28]. Subsequently, however, numerous mutations associated with ADSHE have been identified in various genes other than *CHRNA4*/*CHRNB2,* such as *CRH* [29,30], *KCNT1* [31], *CABP4* [32], *DEPDC5* [33], and *GATOR1* [34], identified from ADSHE pedigrees [35,36,37,38]. In particular, identifications of some mutant genes that do not encode ion channels or are not directly related to acetylcholinergic transmission, such as *CHR*, *DEPDC5,* and *GATOR1*, suggest that ADSHE may be caused by functional abnormalities in a manner other than channelopathy [36]. Additionally, mutations associated with *CRH* residing in the promoter regions have also been identified [29], suggesting that the expression volume of proteins, even those that have no functional abnormalities, might also be involved in the pathomechanisms of ADSHE. Clinical findings indicate that ADSHE/SHE are symptomatically quite similar, but accumulating genetic findings suggest that ADSHE is probably driven by complicated pathomechanisms [36,37,38]. Nevertheless, various genetic abnormalities among ADSHE pedigrees have been identified in *CHRNA4*/*CHRNB2*, and their clinical features caused by mutant *CHRNA4*/*CHRNB2* have also been clearly confirmed. Therefore, ADSHE mutations in *CHRNA4*/*CHRNB2* are referred to as classical mutations and have provided extremely important findings regarding the epileptogenesis and/or ictogenesis of ADSHE [36,37,38].

Initially, functional analyses of ADSHE-mutant nAChRs in transfected single cells, such as *Xenopus oocytes* and human *embryonic kidney* (HEK) cells, using the whole-cell patch–clamp technique, revealed various functional abnormalities in ADSHE-mutant α4β2-nAChRs [39,40,41,42,43,44]. Despite these efforts, these studies could not identify any commonalities associated with functional abnormalities of ADSHE pathomechanisms among ADSHE-mutant nAChRs, including both loss-of-function and gain-of-function [36,37,38]. Subsequently, various genetic rodent models of ADSHE have been developed to analyze epileptogenesis and/or ictogenesis of ADSHE [36,45,46,47,48,49,50,51]. Thus far, although the majority of ADSHE rodent models do not exhibit ADSHE seizures, some strains harboring mutant *Chrna4* (corresponding to human S284L-mutant *CHRNA4*) and *Chrnb2* (corresponding to human V287L-mutant *CHRNB2*) display spontaneous epileptic seizures [47,49,52,53], demonstrating face validity [36]. These two mutations have been energetically investigated for the functional abnormalities associated with ADSHE, including epileptogenesis and ictogenesis, using genetic rodent models [47,49,52,53]. Various preclinical findings regarding functional abnormalities of human S284L-mutant *CHRNA4* and V287L-mutant *CHRNB2* genes have clearly indicated epileptogenesis and/or ictogenesis of these two mutant genes [47,49,52,53]. In particular, functional analyses using ADSHE rat models harboring rat S286L-mutant *Chrna4* (corresponding to human S284L-mutant *CHRNA4*)—namely, S284L-TG and S286L-TG—have demonstrated the complicated age-dependent and event-related functional abnormalities in tripartite synaptic transmission (not only neurotransmission but also gliotransmission), without any brain structural abnormalities in numerous networks [36,47,53,54,55,56,57,58,59,60,61,62,63]. These findings suggest that even if a single gene mutation plays an important role as a primary pathomechanism of ADSHE, the actual epileptogenesis/ictogenesis of ADSHE possibly involves various complicated functional abnormalities in tripartite synaptic transmission, depending on the neurodevelopmental process of transmission [36]. Based on these backgrounds, this narrative review mainly focuses on the developmental process of pathomechanistic networks and tripartite synaptic transmission involved in the age-dependent epileptogenesis and event-related ictogenesis of ADSHE via findings from S284L-TG and S286L-TG.

## 2. Clinical Features of ADSHE/SHE

The first description related to ADSHE dates back to 1968, when it was introduced as a spectrum of “arousal parasomnias” [64]. Subsequently, based on several studies showing that epileptiform discharge, a frontal lobe focus, and mutation in the *CHRNA4* gene were identified in some patients, the spectrum of conditions—including nocturnal paroxysmal arousal (NPA), nocturnal paroxysmal dystonia (NPD), and episodic nocturnal wandering (ENW)—was collectively termed nocturnal frontal lobe epilepsy (NFLE) [1,2]. In 2014, the “nocturnal frontal lobe epilepsy” (NFLE) denomination was changed by an international consensus conference to “sleep-related hypermotor epilepsy” (SHE) [4]. Therefore, although the terminologies describing NPA, NPD, and ENW are clinically outdated in ADSHE, they remain the main clinical terms to describe the typical seizure manifestations of ADSHE [4,36].

Currently, the classification of ADSHE/SHE is characterized by the International League Against Epilepsy: focal epilepsy syndrome with genetic, structural, or genetic–structural etiology; brief focal motor seizures with hyperkinetic or asymmetric tonic/dystonic posturing; and occurring predominantly during non-rapid eye movement (non-REM) sleep [3]. Clinical manifestations between the autosomal-dominant form (ADSHE) and the sporadic form (SHE) are indistinguishable, since they are comparable to frontal lobe epileptic seizures [1,2,3,4]. ADSHE seizures are sleep-related, stereotyped hypermotor seizures consisting of vigorous hyperkinetic features or asymmetric dystonic/tonic features. Patients with ADSHE/SHE usually experience a cluster of hypermotor seizures during the same night [1,2,3,4]. Brief seizures may sometimes evolve into secondarily generalized tonic–clonic seizures [3,4]. Even if ADSHE seizures are controlled for long periods, once a patient experiences an ADSHE seizure, they can experience clustering and many/frequent ADSHE seizures during the same night [1,2,3,4].

Mean onset age of ADSHE/SHE is approximately 10 years, and 80% of individuals develop ADSHE by 20 years of age [3,4,41]. The onset age is one of the important factors for the diagnosis of epilepsy syndromes, so the clinical perspective also suggests that age-dependent (or neurodevelopmental) abnormalities play important roles in development of epileptogenesis and/or ictogenesis of each epilepsy syndrome [36,65,66]. ADSHE/SHE can be classified into two subclasses based on antiseizure medication (mainly carbamazepine) sensitivity and cognitive dysfunction other than genetic features [4,36,41]. Approximately 60–70% of ADSHE/SHE patients can achieve remission and exhibit improved prognosis with relatively low doses of carbamazepine [1,41] while over 30% do not, including those with S284L-mutant *CHRNA4* [1,9,10,14,67]. Approximately 15–45% of patients with ADSHE/SHE have cognitive impairments, especially dysfunction of executive function and verbal IQ, suggesting that cognitive dysfunction is an integral part of ADSHE/SHE caused by heterozygous pathogenic variants in nAChRs in the thalamocortical pathway or frontal lobe [36,68,69]. Indeed, ADSHE patients with S284L-mutant and insL-mutant *CHRNA4* and I312M-mutant *CHRNB2* present disturbance of neuropsychiatric development or schizophrenia-like psychosis [7,10,11,12,14,15,18,70]. Finally, the most distinctive clinical feature of ADSHE/SHE is the arousal/awakening synchronized with ADSHE seizures, since the majority of focal epilepsies—including typical limbic seizures—are generally accompanied by a loss of consciousness [71].

## 3. Phenotypic Features of ADSHE Rodent Models and Validations

The development of conventional antiseizure medications through screening using traditional seizure models, such as kindling, maximal electroshock, and pentylenetetrazol, has undoubtedly increased therapeutic options; however, growing concerns have emerged regarding the efficacy of third-generation antiseizure medications in the treatment of focal epilepsies, as their effectiveness was evaluated to be comparable to those of first- and second-generation drugs [72,73]. In particular, the majority of epileptologists have apprehension that screening strategies based on models lacking epileptogenesis or ictogenesis may fail to identify compounds with actual therapeutic potential [72,73,74]. Paradigm shifts in novel antiseizure medication discovery have recently emphasized the importance of identifying compounds that interfere with epileptogenesis and/or ictogenesis. In this context, therefore, rigorous adherence to validity criteria, including face validity, construct validity, and predictive validity, has become essential in the evaluation of genetic epilepsy rodent models [36,47,53,72,73,74,75,76,77,78,79,80,81]. The evaluation of the genetic ADSHE rodent models in accordance with the established validation criteria has been discussed in previous reports [35,36,82,83,84].

### 3.1. DEPDC5

The *DEPDC5* mutation is currently considered to be one of the most impactful pathogeneses of inherited focal epilepsy, including not only ADSHE but also other genetic focal epilepsy syndromes. Recent clinical studies have reported *DEPDC5* mutations in 3.9–13% of patients with ADSHE/SHE [85,86], with approximately 9% of pedigrees with the *DEPDC5* mutation being diagnosed with ADSHE [87]. However, *DEPDC5*-related epilepsy encompasses a wide range of focal epilepsy syndromes with foci in almost all discrete regions of the cortex, including not only ADSHE but also familial focal epilepsy with variable foci (FFEVF) [88], familial mesial temporal lobe epilepsy (FMTLE) [89], autosomal-dominant epilepsy with auditory features (ADEAF) [89], and approximately 20% of cases involving cortical malformations, typically focal cortical dysplasia (FCD) type II or hemimegaloencephaly [90,91]. Although the majority of individuals with the *DEPDC5* mutation display normal neuropsychiatric development, severe neuropsychiatric comorbidities have been reported [87]. Over 60% of ADSHE patients with the *DEPDC5* mutation are antiseizure medication-resistant [88]. *DEPDC5* forms a GATOR1 complex with NPRL2 and NPRL3, which inactivates mTORC1 [92]. Considering the signaling function of *DEPDC5*, it is reasonable that loss-of-function of *DEPDC5* is involved in a wide range of focal epilepsies. These findings suggest that although *DEPDC5* mutations may be involved in various focal epilepsies, they may not be suitable for elucidating the pathomechanisms of typical ADSHE, which is not accompanied by brain structural abnormalities. Indeed, *DEPDC5*-mutant rodent models exhibit various brain structural abnormalities but have not achieved phenotypes (ADSHE seizure) that fulfill face validity for the ADSHE model [93,94,95].

### 3.2. CHRNB2-Mutant Models

ADSHE patients with the V287L-mutation exhibit typical ADSHE seizures, mainly in the non-REM sleep phase. Although the ADSHE features are relatively homogeneous among families, seizure severity and specific frontal lobe seizure manifestations vary within families [25]. For some individuals, ADSHE seizures are either so mild that medical attention is not sought or are easily controlled by carbamazepine, whereas others indicate carbamazepine resistance [25]. Neuropsychiatric comorbidities in ADSHE patients with the V287L-mutation, including psychiatric disorders or disturbance of neuropsychiatric development, have not been reported [20,25,96]. V287L-mutant nAChRs are considered to be gain-of-function, since increasing ACh sensitivity with peak currents and delayed desensitization of V287L-mutant nAChRs have been observed [20,25,49,52]. To date, three rodent models carrying mutant *Chrnb2* corresponding to human V287L-mutant *CHRNB2* have been engineered. A knock-in mouse model did not display spontaneous seizures but showed enhanced sensitivity to seizures induced by nicotine [51]. A transgenic rat model, V286L-TG, showed spontaneous epileptic seizures, such as NPA and ENW (onset was approximately 8 weeks of age), but NPD was not observed [52]. V286L-TG also showed enhancement of sensitivity to seizures induced by nicotine [52]. Increasing sensitivity of these two models to nicotine is supported by the functional abnormalities, such as increasing sensitivity and gain-of-function of V287L-mutant nAChRs [20,36,43,52,84]. The third model, β2-V287L, employing the TET-OFF conditional expression system (carrying one to four copies of mutant transgenes), displayed gene-dosage-dependent interictal spikes and spontaneous epileptic seizures [49]. The background EEGs of ADSHE patients are usually comparable to those of healthy controls, but the background EEGs of β2-V287L are dominated by delta-waves. Most seizures are observed during periods of increased delta activity. Importantly, silencing the mutant transgene expression after the onset of ADSHE seizures does not reverse the established epileptic seizure [49]. Furthermore, chronic administration of carbamazepine (30 mg/kg) does not improve epileptic seizures in β2-V287L, which has been shown to be highly effective in in vitro experiments [48,49].

Although no consistent phenotype has been observed among these V287L-mutant rodent models, β2-V287L (carrying four copies of mutant transgenes) shows strong seizures, but V286L-TG (carrying one copy) displays mild epileptic seizures [48,49,52]. Importantly, silencing V287L-mutant *Chrnb2* in β2-V287L does not suppress epileptic seizures [49]. These findings suggest that V287L-mutant *CHRNB2* is involved in the epileptogenesis but not ictogenesis of ADSHE.

### 3.3. CHRNA4-Mutant Models

To date, there are three *CHRNA4*-mutant rodent models, harboring mutant *Chrna4* gene corresponding to human S280F-, insL- and S284L-mutant *CHRNA4* genes. S280F-mutant models (previously designated “S248F” according to reference sequence NP_000735.1) were generated in two mouse strains and named S252F-KI and S248F-KI [45,46]. The insL-mutant *CHRNA4* model was generated in one mouse strain, named insL-KI [45]. S284L-mutant models (previously designated “S252L” according to the reference sequence NP_000735.1) generated two rat strains, named S284L-TG and S286L-TG [47,53].

#### 3.3.1. S280F- and insL-Mutant Models

S280F-mutant and insL-mutant α4β2-nAChRs enhance ACh sensitivity and display use-dependent potentiation [39,40,97]. The identical functional abnormalities among S280F-mutant and insL-mutant nAChRs, with a combination of enhanced ACh sensitivity and use-dependent potentiation, are considered to be enhanced functions of the excitatory cation channel [26]. In spite of different mutations, S252F-KI and insL-KI displayed similar phenotypes; however, although S280F-KI and S252L-KI have the same mutation, the phenotypic features between these models were quite different [45,46]. Both S252F-KI and insL-KI mouse models indicated increasing slow waves (0.5–4 Hz) in backgrounds and spontaneous epileptic discharges during wakefulness, whereas neither spontaneous epileptic seizures nor EEG background abnormalities were observed in S248F-KI [45,46]. Therefore, these indicated phenotypes among these three *CHRNA4*-mutant model mice could not verify the face validity [36,45,46,84].

#### 3.3.2. S284L-Mutant Models

The phenotypic features of two transgenic rat models, harboring S286L-mutant rat *Chrna4*, corresponding to human S284L-mutant *CHRNA4*, named S284L-TG and S286L-TG, have been confirmed [47,53]. Both models show no background abnormalities in EEGs and exhibit spontaneous ADSHE seizures, such as ENW, NPA, and NPD, during non-REM sleep [36,47,53]. However, the seizure frequencies in these models were considered lower than those in untreated ADNFLE patients due to the inability to observe clustering [47,57]. In the initial studies of S284L-TG and S286L-TG, and NPA in S284L-TG and S286L-TG was detected electro-physiologically as very brief (within a second) and intense motor episodes (eye opening with sudden jumping or running) accompanied by arousal/awakening, though they occurred very infrequently—approximately once every few months [47,53]. A similar phenotype to this type of NPA in S284L-TG/S286L-TG was observed in another ADSHE genetic rat model (V286L-TG) [52]. In these three studies, polyspikes lasting several seconds and not accompanied by movements were counted as interictal discharge [47,52,53]. However, in a recent study, video-monitored EEGs showed that polyspikes without movements could be classified into two types: those without and those with eye-opening behaviors [63]. Thus, polyspikes with eye opening (without other movements) were re-defined as NPA [63], which indicates the typical features of NPA for patients with ADSHE [1,2,98,99]. This re-definition (open eyes with polyspikes as NPA) is supported by the previous clinical and pharmacological findings that the polyspike frequencies of S284L-TG and S286L-TG are decreased by zonisamide and benzodiazepine but not by carbamazepine [47,53,57,63], similar to NPA in ADSHE patients with the S284L-mutation [9,10,14,36,91]. Therefore, the seizure frequency of S286L-TG, which is carbamazepine-resistant and zonisamide-sensitive, is over 20 per day with clustering—comparable to that of untreated patients with ADSHE [1,2,53,60,100].

The phenotypic features (face validity) and antiseizure medication responses (predictive validity) between S284L-TG and S286L-TG are quite similar [36]. The expression of wild-type versus S286L-mutant *Chrna4* in S284L-TG has been shown to be 45% versus 55%, with the total expression of α4-nAChRs being almost equal between the wild-type and S284L-TG [47]. Although the constitutive validity of S284L-TG has been confirmed in terms of transgene expression, the promoter in S284L-TG was adopted from the rat PDGF-β promoter [47]. Therefore, while the transgene expression in S284L-TG is ideal, the expression-regulating system of the PDGF-β promoter poses a major concern in potential drug discovery research. In other words, the pharmacological validity [36] of S284L-TG cannot be guaranteed. Based on these limitations, the promoter in S286L-TG was adopted for the rat natural *Chrna4* promoter [53]. The expression of wild-type versus S286L-mutant *Chrna4* in S286L-TG was shown to be 60% versus 40%, with the total expression of α4-nAChRs being almost equal between the wild-type and S286L-TG [53].

S284L-TG is confirmed as an ADSHE model, demonstrating face, construct, and predictive validities. S286L-TG is confirmed to be an ADSHE model as well, demonstrating pharmacological validity in addition to face, construct, and predictive validity.

## 4. Transmission Abnormalities in S284L-TG and S286L-TG

**Figure 1:**
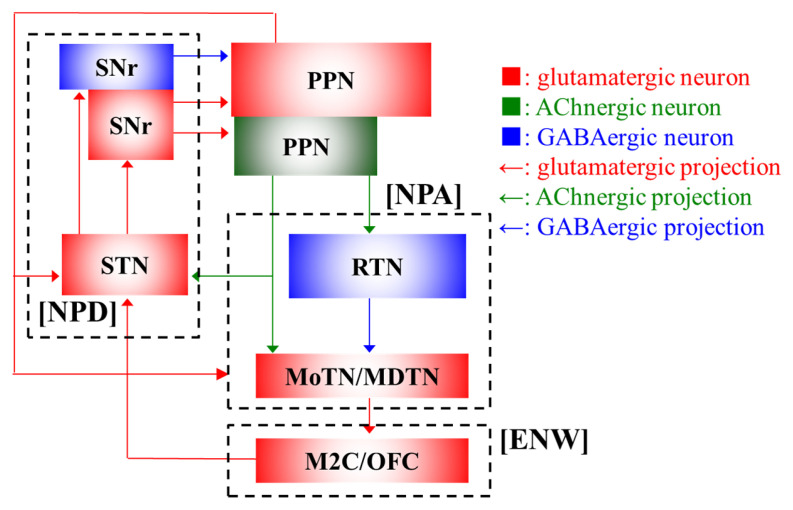
The pedunculopontine nucleus (PPN) is one of the established nuclei of acetylcholinergic neurons in the mesencephalic locomotor region [101,102,103]. In the PPN, at least two types of neurons, such as glutamatergic and acetylcholinergic neurons, co-existed [102,103,104]. The PPN has been considered to be involved in various functions, including sleep/wakefulness, cognition (attention, learning, and reward), and movement/locomotion [102,103] via projections to various regions. Acetylcholinergic neurons in the PPN project to the GABAergic neurons (in the reticular thalamic nucleus: RTN) and glutamatergic neurons (motor thalamic nuclei: MoTN and mediodorsal thalamic nucleus: MDTN) in the thalamus [105,106,107], and glutamatergic neurons in the basal ganglia (mainly subthalamic nucleus: STN) [106]. Glutamatergic neurons in the PPN also project to the glutamatergic neurons in the basal ganglia (STN) [108,109] and the thalamus (MoTN and MDTN) [105,110]. GABAergic neurons in the RTN project to other glutamatergic nuclei in the MoTN and MDTN [111,112], and those in the SNr project to glutamatergic neurons in the PPN [113,114]. Glutamatergic neurons in the thalamus project glutamatergic terminals to various cortices, including secondary motor cortex (M2C) and orbitofrontal cortex (OFC) [53,55,57,61,115]. STN receives various glutamatergic projections from M2C and MoTN [116,117], and projects glutamatergic terminals to glutamatergic and GABAergic neurons in the substantia nigra pars reticulata (SNr) [118]. Both glutamatergic and GABAergic neurons in the SNr project their terminals to glutamatergic and acetylcholinergic neurons in the PPN [114,119,120]. Typical ADSHE seizures, EEG-sensitive nocturnal epileptic wandering (ENW), and nocturnal paroxysmal arousal (NPA) are propagated from the frontal cortex and thalamus, respectively, whereas EEG-insensitive nocturnal paroxysmal dystonia (NPD) is a hyperactivation in the basal ganglia, consequently being clinically EEG-insensitive. Red, green, and blue arrows indicate the projections of glutamatergic, acetylcholinergic, and GABAergic terminals in S286L-TG, confirming that after the onset of ADSHE seizures (over 8 weeks of age), they can be detected using microdialysis, respectively.

In order to explore the epileptogenesis and/or ictogenesis underlying focal epilepsy, which has no structural abnormalities, it is necessary to predict target networks/circuits for functional analysis as accurately as possible based on data from electrophysiological examination and seizure phenotypes [36]. Especially, precision of selecting target networks/circuits based on seizure phenotypes critically affects the provision of evidence for subsequent analyses of ADSHE pathomechanisms, since ADSHE has EEG-sensitive ENW and EPA, and EEG-insensitive NPD. Based on the phenotypic features observed in S284L-TG and S286L-TG, the predicted networks/circuits for functional analyses are represented in Figure 1.

Fundamental phenotypic feature of ADSHE seizures, which are composed of three types—motor seizures with epileptic focus in the primary/secondary motor cortex (ENW) [47,53], EEG-insensitive arousal with dystonic posturing (NPD) with probably functional abnormalities in basal ganglia [57], and arousal with eye opening and polyspikes in EEG (NPA) during the non-REM phase—highlight the networks containing the pedunculopontine tegmental nucleus (PPN) and its projection regions, including thalamus, basal ganglia and frontal cortex [53,55,57,60,61,62,63,102,121,122,123]. Additionally, α4β2-nAChRs are widely expressed in the central nervous system, including in the thalamus, basal ganglia, and frontal cortex [124,125,126]. Therefore, the acetylcholinergic, glutamatergic, and GABAergic pathways among PPN, thalamus, basal ganglia, and the frontal cortex as key nodes in regulating the sleep–wake cycle, dystonic posturing, and motor initiation are the potential targets for exploring the pathomechanisms of ADSHE [102,121,122,123]. Indeed, the PPN projects both acetylcholinergic and glutamatergic terminals to the thalamus (meso-thalamic pathway) and the basal ganglia (extrapyramidal pathway) and receives GABAergic terminals from the substantia nigra reticulata (SNr) [106,107,108,110,111,113,118,119,120]. Taken together, these findings suggest that the interplay among acetylcholinergic, glutamatergic, and GABAergic signaling within the PPN-centered networks may play pivotal roles in the generation and modulation of ADSHE-related seizure phenotypes. Future studies targeting these pathways may offer deeper insights into the neurophysiological substrates of non-lesional focal epilepsy and inform the development of phenotype-specific therapeutic strategies.

To analyze the functional abnormalities in model animals that fulfilled the validity criteria, the first step involves characterizing the pharmacological properties of the loss-of-function S286L-mutant nAChRs in S286L-TG to clarify the molecular and functional abnormalities in targeted networks/circuits of rodent models. Subsequently, comparing the synaptic transmission abnormalities during the interictal stages between S286L-TG and wild-type can provide the actual transmission abnormalities during the interictal stage and can provide detailed maturation process of epileptogenesis or ictogenesis. Such findings are critical for therapeutic development: they enable the design of targeted interventions that either modulate the maturation of epileptogenic circuits or directly suppress seizure initiation. This distinction lays the foundation for a more precise drug discovery strategy, potentially leading to novel treatments tailored to the specific phase of epileptogenesis affected by the mutant genes [36,84].

### 4.1. Transmission Abnormalities During the Interictal Stage, Including Wakefulness and SWS

Activity of acetylcholinergic neurons in the PPN decreases during the transition from wakefulness to the slow-wave sleep phase (SWS) [127], resulting in decreased extracellular ACh levels in the PPN and its projection regions [63] (Table 1). In S286L-TG, extracellular ACh levels in the PPN, MoTN/MDTN, M2C/OFC, STN, and RTN are generally equivalent to those in wild-type during both wakefulness and SWS (Table 2). Therefore, S286L-TG has congenitally impaired α4β2-nAChRs, whereas the functional abnormalities in the regulation mechanisms of ACh release (ACh exocytosis) in S286L-TG after the onset of ADSHE cannot be detected, suggesting that downstream transmission systems regulated by α4β2-nAChRs, rather than acetylcholinergic transmission, are probably fundamental functional abnormalities for epileptogenesis or ictogenesis of S286L-TG.

PPN projects acetylcholinergic terminals to the thalamus, including RTN and MoTN/MDTN [105,106,107] (Figure 1). Basal extracellular GABA levels in the RTN, MoTN/MDTN, and PPN of S286L-TG are almost equivalent to those in wild-type [47,53,55,57,61,63] (Table 2). However, in wild-type, extracellular GABA levels in the RTN and MDTN/MoTN decrease during the transition from wakefulness to SWS, whereas in S286L-TG, extracellular GABA levels during SWS and wakefulness are almost equal [36,47,63,128] (Table 1). On the contrary, in both S286L-TG and wild-type, extracellular GABA levels are equivalent between wakefulness and SWS in the M2C/OFC and STN, which do not directly receive acetylcholinergic projection from the PPN [55,56,57,63] (Figure 1 and Table 1). Therefore, influence of GABAergic disinhibition was observed in the thalamus, which received acetylcholinergic projection from the PPN, in S286L-TG (compared to wild-type).

After the onset of ADSHE seizures (older than 8 weeks of age) during both wakefulness and SWS, S284L-TG and S286L-TG consistently display higher basal extracellular L-glutamate levels in the PPN, RTN, MoTN/MDTN, STN, SNr, and M2C/OFC compared to wild-type [47,53,55,57,63] (Table 2). In wild-type, extracellular L-glutamate levels in the PPN, MoTN/MDTN, STN, SNr, and M2C/OFC decrease during SWS compared to wakefulness [47,53,55,57,63], whereas the physiological reduction in extracellular L-glutamate levels in these regions is not observed in S286L-TG or S284L-TG [47,53,55,57,63] (Table 1). Contrary to this, in both S284L-TG and S286L-TG, basal extracellular L-glutamate levels in the OFC/M2C, MDTN/MoTN, and STN are comparable to those of wild-type at 4 weeks of age (before onset of ADSHE seizure or interictal discharge) [47,53]. These results suggest that enhanced glutamatergic transmission in S286L-TG compared to wild-type becomes further enhanced during SWS. Therefore, increasing glutamatergic tone during SWS is possibly involved in the event-related (sleep-related) ictogenesis of ADSHE seizures of S284L-TG and S286L-TG.

### 4.2. Impact of Loss-of-Function of S286L-Mutant α4β2-nAChRs in S286L-TG

ADSHE-mutant α4β2-nAChRs exhibit enhancement of sensitivity to ACh and desensitization, but other common parameters among ADSHE-mutant α4β2-nAChRs could not be detected [39,40,43,44,53]. Extracellular Na^+^ and Ca^2+^ inflows through α4β2-nAChRs play important roles in the regulation of depolarization, exocytosis, and intracellular signaling [40,43,53]. The reduced Ca^2+^ permeability of S280F-, insL- and V287L-mutant α4β2-nAChRs is an impairment, but is not observed in S286L-mutant α4β2-nAChRs [7,43]. However, S286L-mutant α4β2-nAChRs enhanced the reduction in the total Ca^2+^ inflow due to enhanced desensitization [43,44]. Based on these findings, the abnormalities observed in human S284L-mutant α4β2-nAChRs are likely due to loss-of-function.

In wild-type, local administration of selective α4β2-nAChR agonist (RJR2403) into the M2C and OFC using the reverse dialysis method increased extracellular levels of both L-glutamate and GABA in these regions [53]. Local administration of RJR2403 into the MoTN and MDTN also increased extracellular levels of both L-glutamate and GABA in these regions [53], as well as increased extracellular L-glutamate levels in the M2C and OFC [53]. The activation of acetylcholinergic neurons in the PPN activates GABAergic neurons in the RTN via α4β2-nAChRs, leading to the propagation of GABAergic inhibition to thalamic glutamatergic neurons [100]. On the contrary, in S286L-TG, stimulatory effects of α4β2-nAChRs on the release of L-glutamate and GABA in the frontal cortex and thalamus were attenuated compared to the wild-type [53]. In S286L-TG, local administration of RJR2403 into the RTN increased the extracellular L-glutamate levels in the MoTN/MDTN and STN [53,55,56,57]. These results suggest that impaired S286L-mutant α4β2-nAChRs in S286L-TG attenuate the activation of both glutamatergic and GABAergic transmissions, but that the attenuated S286L-mutant α4β2-nAChRs predominantly affect the inhibitory function of GABAergic transmission rather than excitatory glutamatergic transmission, resulting in relatively enhanced excitatory transmission in S286L-TG [36].

Interestingly, neither the M2C nor OFC can generate epileptic discharge independently; however, they can integrate external excitatory inputs from the thalamocortical pathway, resulting in the generation of epileptic discharge in these regions [55,56,57,58]. The hyperactivation of the thalamo-subthalamic pathway suggests that propagation of hyperactivation of the MoTN in S286L-TG to predominantly the STN rather than the frontal cortex generates EEG-insensitive tonic/dystonic or hyperkinetic behavior, such as NPD [57]. Despite these efforts, increased basal extracellular L-glutamate levels in the MoTN/MDTN, STN, and M2C/OFC of S286L-TG cannot be fully explained by intra-thalamic GABAergic disinhibition, as intra-thalamic GABAergic transmission is phasic inhibition [53].

### 4.3. Transmission Abnormalities During ADSHE Seizures

In S284L-TG, extracellular levels of L-glutamate and GABA in the frontal cortex drastically increase during ENW, while after ENW resolution, they recover to the levels before ENW onset [47]. The increase in GABA level associated with ENW and recovery after ENW resolution is delayed compared to L-glutamate [47]. In S286L-TG, during NPA, extracellular levels of L-glutamate and ACh in the PPN and its projection regions (MoTN, STN, SNr, and RTN) drastically increase compared to SWS before NPA onset [63]. In S286L-TG, during NPD (EEG-insensitivity with motor events), extracellular levels of L-glutamate and ACh in the MoTN and STN also drastically increase. After NPA and NPD resolution, increasing extracellular levels of L-glutamate and ACh during NPA and NPD recover to values before the ADSHE seizures. However, increasing L-glutamate levels persist after NPD compared to NPA in the same individuals, whereas ACh levels rapidly recovered in the same way after NPA and NPD [63].

## 5. Astroglial Age-Dependent Epileptogenesis and Event-Related (Sleep-Related) Ictogenesis in S286L-TG

The transmission abnormalities in epileptic foci or epileptogenic circuits related to symptoms of ADSHE seizure include an imbalance between GABAergic and glutamatergic transmission during non-REM sleep, consistent with the traditional pathophysiology hypothesis of epileptic seizures [26,36]. However, while this imbalance alone can partially explain the event-related (sleep-related) ictogenesis of ADSHE seizures, it cannot fully explain the age-dependent development of epileptogenesis underlying the clinical features of ADSHE seizures.

It has been shown that the ADSHE onset of S284L-TG can be partially prevented by chronic administration before ADSHE onset of the NKCC1 inhibitor, furosemide [54]. This experiment was based on the finding of upregulation of NKCC1 after ADSHE onset in the frontal cortex of S284L-TG, which was not observed before ADSHE onset [54]. The other pharmacodynamic target of furosemide is known to be the inhibition of mitogen-activated protein kinase and extracellular signal-regulated kinase (MAPK/Erk) [129]. MAPK/Erk signaling regulates protein phosphorylation and cell functions, such as proliferation, division, differentiation, survival, and apoptosis [58,59,130,131]. These demonstrations suggested that the development of ADSHE epileptogenesis involves interactions among various factors that cannot be assumed solely based on factors directly related to the mutant gene [36].

Basal extracellular L-glutamate levels constantly increase during the development of epileptogenesis in several regions, where α4β2-nAChRs are expressed, showing distinctive functional abnormalities compared to other transmission systems in S286L-TG and S284L-TG [36,47,53,55,57,60,61,62,63]. The lack of decreasing L-glutamate release during SWS cannot be explained by acetylcholinergic transmission and S286L-mutant α4β2-nAChRs function. Additionally, a specific clinical feature of ADSHE—that even in patients whose seizures have been controlled for a long time, it is not uncommon for frequent seizures or clustering to be observed in the same night once a seizure occurs [1,2,3,4]—suggests that some molecular mechanisms that accumulate epileptogenic excitabilities may be involved in ADSHE ictogenesis. Considering these clinical features of ADSHE, it is not surprising that astroglial hemichannels have been identified as candidate molecules that may be upregulated by Erk signaling and cause a persistent increase in L-glutamate release [32], since astroglial hemichannels remain activated for several hours [36,132]. It is well-known that hemichannels are composed of various molecules, such as connexin and pannexin families [133,134].

### 5.1. Impact of Upregulated Connexin43 in S286L-TG

Hemichannel plays important roles in astrocytes as a regulatory molecule, mediating biochemical communication both between adjacent cytoplasmic cells (gap junctions) and between the intracellular and extracellular space (via hemichannels). A connexon is formed by six homomeric and/or heteromeric connexins, and a single connexon mediates communication between the intra- and extracellular spaces (hemichannel), whereas two opposed connexons form a gap-junction, enabling cytoplasm–cytoplasm communication [135,136,137]. Specific functional features of both hemichannels and gap-junctions are emphasized: They provide communication for molecules of a wide range of molecular weights, ranging from low (i.e., cations) to high (i.e., eicosanoids and ATP) [135,138]. Hemichannel permeability is persistently activated by hyperactivation (over several hours), such as depolarization, ischemia, specific cation mobilization, and phosphorylation, whereas gap-junction permeability remains stable at resting membrane potentials [135,138]. Therefore, activation of hemichannels plays important roles in the non-exocytotic release of excitatory astroglial transmitters, including L-glutamate, D-serine, ATP, kynurenine metabolites, and eicosanoids, leading to the disruption of homeostasis [135,138]. Importantly, hyperexcitable tripartite synaptic transmission associated with upregulated/activated connexin43-hemichannels promotes the persistent (lasting several hours) astroglial release of excitatory gliotransmitters, such as L-glutamate, D-serine [61], and ATP [61,62], which likely contribute to an imbalance in transmission, thus possibly promoting ictogenesis [36].

In the OFC of neonatal and 4-week-old (before the occurrence of interictal discharge) S286L-TG, upregulation of Erk signaling is observed, but neither connexin43 expression nor Akt signaling is increased [58,61,62]. Chronic administration of nicotine downregulated Erk signaling in wild-type but did not affect Erk signaling in S286L-TG [58]. Therefore, loss-of-function of S286L-mutant nAChRs in S286L-TG possibly also results in an inhibitory effect of Erk signaling [58].

On the contrary, in S286L-TG over 8 weeks of age (after ADSHE onset), expression of connexin43 and signaling of Akt and Erk are increased compared to those in wild-type. Chronic nicotine administration decreased Erk signaling in wild-type; however, these suppressive effects of nicotine on Erk signaling were not observed in S286L-TG before and after the ADSHE onset period [58]. Therefore, S286L-mutant α4β2-nAChRs reduce not only their cation channel function (inflow cation) but also their suppressive effects on Erk signaling [58]. These findings suggest that a combination of increasing Erk signaling and GABAergic disinhibition induced by loss-of-function of S286L-mutant α4β2-nAChRs plays important roles in the initial stage of development of epileptogenesis in S286L-TG [58,60,61,62].

### 5.2. Impact of Upregulated Pannexin1 in S286L-TG

Upregulation of connexin43 alone cannot fully explain the increased basal L-glutamate level in epileptogenic regions in S286L-TG, as connexin43-hemichannels generally do not function during the resting stage due to their lower opening probability [139,140,141,142]. In contrast to connexin43-hemichannels, the threshold membrane potentials for pannexin1-hemichannels are around the resting membrane potential [21,143]. Indeed, after ADSHE onset, abnormalities in exocytosis and reuptake of L-glutamate in S286L-TG cannot be detected [36], whereas the expression of pannexin1 in the plasma membrane of the OFC of S286L-TG increases compared to the wild-type [61]. Upregulated pannexin1 is a potential molecular candidate for increasing basal L-glutamate release in the epileptogenic region of S286L-TG. Pannexin1 and connexin43 do not share homologies in their peptide sequences, whereas connexin43 and pannexin1 are assembled in hexamers to form connexon and pannexon in the plasma membrane, respectively [135,136,144]. The topologies and structures in the proteins of connexon and pannexon comprise a similar group of transmembrane pores, which are permeable to ions, second messengers, and several signaling mediators up to 1.5 kDa [145,146]. Therefore, both connexon and pannexon serve as routes of ionic and large molecular interchange between the cytoplasm and extracellular compartment [135,136]; however, pannexon is considered likely unable to constitute a gap-junction [147]. Furthermore, pannexin1-hemichannels can open due to their lower negative threshold around resting membrane potential [139,140]. Therefore, both connexin43- and pannexin1-hemichannels are activated by depolarization, but pannexin1-hemichannel can reach maximum currents with faster kinetics [139,143]. Furthermore, the gating properties of connexin43-hemichannel are regulated by extracellular Ca^2+^ dependency (the physiological range of the extracellular Ca^2+^ level inhibits the probability of connexin43-hemichannel opening); however, pannexin1-hemichannel opening is independent of the extracellular Ca^2+^ level and is instead activated by increasing intracellular Ca^2+^ level [139,143].

### 5.3. Impact of High-Frequency Oscillation in S286L-TG

Wide-band EEG frequently detects the synchronization of high-frequency oscillation (HFO) bursts with slow direct-current shift (DC-shift) in the epileptogenic zone prior to epileptic seizure onset [148,149,150]. Interaction/synchronization of HFO with DC-shift is considered to play important roles in ictogenesis [148,149,150]. HFO—which clearly stands out from the baseline and persists for at least four oscillation cycles—is composed of two frequency ranges: relatively slow physiological ripple (80–250 Hz and duration of tens of milliseconds) and epileptogenic fast ripple (250–500 Hz and duration of milliseconds) [148]. Experimentally, HFO is induced by reducing extracellular Ca^2+^ with increasing K^+^ levels [151]. This extracellular cation condition is also observed during or immediately after epileptic seizures [152]. Although sleep spindles are generally considered to be coupled with physiological ripple-HFO [153,154,155], several reports have speculated that sleep spindles play important roles in the ictogenesis of ADSHE, since the onset of ADSHE seizures after sleep spindle is not clinically uncommon [1,4]. Indeed, in rodent epilepsy models (kindling and PTZ-induced models), HFO has been recorded in the focal region prior to seizure onset [156,157]. Therefore, ripple-HFO, which has no pathological effects in healthy individuals, is probably involved in the ictogenesis of patients with acquired ADSHE epileptogenesis. On the contrary, duration of DC-shift is usually longer than tens of seconds [149,158]. Traditionally, DC-shifts are considered to be generated by increasing extracellular K^+^ concentration due to functional abnormalities in K^+^ buffering of astrocytes [159]. Therefore, it is possible that HFO induced by DC-shift or overlapping HFO propagation further contributes to ictogenesis. As expected, during the preparatory period of ADSHE onset (during 6–8 weeks of age), synchronized ripple-HFO and interictal discharge can be detected [60]. Even after the onset of ADSHE, synchronized ripple-HFO with interictal discharge can also be detected [60]. Notably, a series of electrophysiological events can be detected, during which ictal discharge occurs after the transition from ripple-HFO to fast-ripple-HFO or partially following sleep spindles [60].

Recently, several preclinical studies reported that HFO and hemichannels have a mutually reinforcing interaction. In vivo experiments using a pilocarpine-induced epilepsy model demonstrated increasing spectral power of HFO, which was inhibited by a connexin43-hemichannel inhibitor, carbenoxolone [160,161]. In vitro experiment demonstrated that artificial fast-ripple-HFO evoked stimulation-activated astroglial transmitter release, which was suppressed by inhibitors of connexin43-hemichannels (Gap19 and carbenoxolone) and pannexin1-hemichannels (10PANX and probenecid); however, the stimulatory effects of artificial ripple-HFO on astroglial transmitter release were quite weak, but long-term exposure to ripple-HFO increased expression of connexin43 and pannexin1 in the plasma membrane [57,61,62].

In the OFC of neonatal S286L-TG, increasing Erk signaling can be detected, but connexin43 expression or increasing Akt signaling cannot be detected [60], whereas Erk signaling and connexin43 expression in cultured astrocytes (after a 28-day culture) are almost equal between S286L-TG and wild-type. This discrepancy of Erk signaling between ex vivo and in vitro experiments suggests that upregulated Erk signaling in S286L-TG is reversible [60]. In other words, increasing Erk signaling disappears during 28 days of culture. Acute artificial HFO-evoked stimulation frequency dependence increases astroglial L-glutamate release when the electrical quantity is set the same (impact of fast-ripple-evoked stimulation is greater than ripple-evoked stimulation), without affecting the expression of connexin43 in the plasma membrane [60,61,62]. On the contrary, chronic artificial HFO-evoked stimulation increases astroglial L-glutamate release and the expression of both connexin43 and pannexin1 in the plasma membrane via increasing Erk signaling [58,61,62]. Therefore, HFO acutely activates hemichannel activity without affecting its expression, but chronically activates not only the activity but also the expression of connexin43 and pannexin1 in the plasma membrane via activation of Erk signaling [60,61,62]. These findings support the possibility that physiological ripple-HFO during non-REM sleep contributes to the development of epileptogenesis over time. Indeed, ex vivo experiments have highlighted the age-dependent increasing expression of connexin43 and pannexin1 in the frontal cortex of S286L-TG [58,61].

### 5.4. Impact of Upregulated P2X7R in S286L-TG

In the S286L-TG rat model, P2X7R expression is enhanced in the OFC, identified as a major ADSHE focal region [62]. This enhancement displays age-dependent progression [62]. At 4 weeks of age (before ADSHE seizure onset), no functional abnormalities related to purinergic transmission, including ATP release or P2X7R expression, are observed in the OFC of S286L-TG. However, at 7 weeks of age (the critical period for interictal discharge onset of S286L-TG), P2X7R expression, basal ATP release, and P2X7R agonist-induced ATP release are significantly enhanced in the OFC [62]. This age-dependent increase in ATP release continues to be observed at 10 weeks of age (after ADSHE seizure onset). These findings suggest that the age-dependent increase in ATP release and P2X7R expression in the OFC possibly contribute to the development of epileptogenesis and/or ictogenesis.

The astroglial release of ATP is mainly regulated by connexin43-hemichannels, pannexin1-hemichannels, and P2X7R [162,163]. P2X7R is widely expressed in the brain, with its highest density being in microglia and neurons, and is also expressed in astrocytes [164]. The basal extracellular ATP levels in the OFC of S286L-TG are higher than those in wild-type at 7 weeks of age, but those between wild-type and S286L-TG at 4 weeks of age are almost equivalent [61,62,165]. Therefore, an increase in the astroglial ATP release in the OFC of S286L-TG is generated before the onset of ADSHE seizures. P2X7R expression in the OFC of S286L-TG does not increase at 4 weeks of age (before ADSHE onset); however, at 7 weeks of age (which is the critical period for interictal discharge onset), P2X7R expression in the OFC of S286L-TG significantly increases compared to that in 4-week-old S286L-TG and 7-week-old wild-type [61,62,165]. This enhanced P2X7R expression indicates a similar temporal fluctuation pattern to the upregulation observed for connexin43 and pannexin1.

Perfusion with BzATP (P2X7R agonist) into the OFC increased the extracellular ATP levels in both wild-type and S286L-TG in a concentration-dependent manner. Notably, the response and sensitivity of P2X7R to BzATP were increased in S286L-TG compared to wild-type. Carbenoxolone (non-selective hemichannel inhibitor) did not affect basal or BzATP-evoked ATP releases in wild-type, but in S286L-TG, it decreased basal ATP release and persistently increased the release of ATP after BzATP discontinuation [61,62]. This suggests that the ATP release in S286L-TG is likely regulated by astroglial hemichannels and/or the P2X7R/pannexin1 complex [61,62]. HFO-induced ATP release was also enhanced by sustained exposure to modest levels of BzATP [61,62]. P2X7R is known to form a complex with pannexin1, mediating large pore formation and gliotransmitter release (including D-serine and ATP) [163,166]. BzATP-evoked D-serine release from cultured astrocytes was suppressed by the selective pannexin1-inhibitor 10PANX, but not by the selective connexin43-hemichannel inhibitor Gap19. This suggests the involvement of the P2X7R/pannexin1 complex in D-serine release [61,62].

Long-term exposure (14 days) of cultured astrocytes to a modest concentration of BzATP, which does not acutely increase astroglial ATP release, enhanced the expression of both P2X7R and connexin43 induced by activated signaling of Akt and Erk [61,62]. Akt signaling specifically promotes the expression of P2X7R, while Erk signaling is considered not to directly contribute to P2X7R expression [167,168]. In S286L-TG, phosphorylated Erk (pErk) expression in the OFC plasma membrane is already upregulated at 4 weeks of age (before ADSHE onset) compared to that in wild-type, and this elevated pErk level persists at 12 weeks of age. Phosphorylated Akt (pAkt) levels are similar to wild-type levels at 4 weeks of age but become upregulated at 12 weeks of age in S286L-TG [61,62]. Therefore, increasing P2X7 and Akt signaling likely contributes to the progression of ADSHE or clustering of ADSHE seizures. Upregulation of P2X7R in S286L-TG is composed of complicated processes. It is probably initiated by the functional abnormalities of the S286L-mutant nAChRs, leading to GABAergic disinhibition and early upregulation of Erk signaling. In conjunction with physiological HFO, this drives the age-dependent increase in P2X7R and connexin43 expression via activated Akt/Erk pathways, possibly contributing to the development of epileptogenesis. To further understand the pathomechanisms of ADSHE, both the expression and the function of P2X7R in neurons and microglia should be explored in further studies.

## 6. Potential Medication Targeting Tripartite Synaptic Transmission

**Figure 2:**
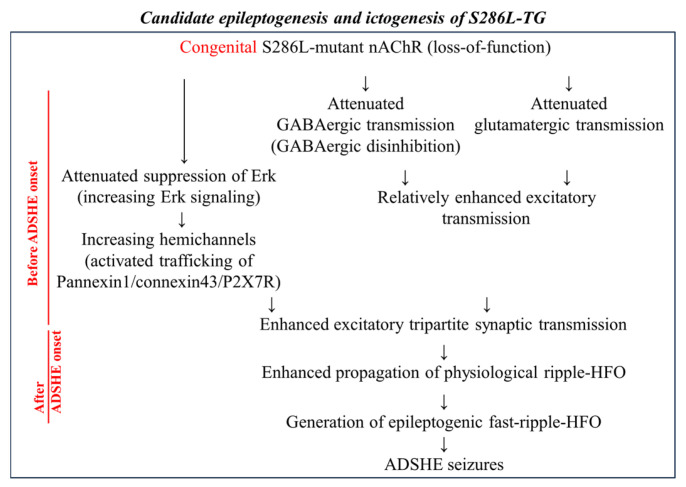
Hypothetical age-dependent epileptogenesis and event-related (sleep-related) ictogenesis of S286L-TG. Congenitally impaired S286L-mutant nAChRs in S286L-TG lead to GABAergic disinhibition and increased Erk signaling [47,53,60,62]. These functional abnormalities in S286L-TG have been observed before the onset of ADSHE seizures. GABAergic disinhibition enhances propagation of physiological ripple-HFO excitability, leading to further increasing Erk signaling during SWS [60,61]. Increased Erk signaling enhances trafficking of pannexin1, connexin43, and P2X7R to the astroglial plasma membrane [60,62]. Physiological ripple-HFO and enhanced Erk signaling activated hemichannels containing pannexin1, connexin43, and P2X7R, consequently generating epileptogenic fast-ripple-HFO. The onset period of interictal and ictal discharges in S286L-TG was approximately 6 and 8 weeks of age, respectively [36,53]. During this preparatory period of ADSHE onset (during 6–8 weeks of age), synchronized ripple-HFO and interictal discharge were observed, but fast-ripple-HFO could not be detected [60]. After the onset of ADSHE (8 weeks of age), a series of electrophysiological events, ictal discharge, occurred after the transition from ripple-HFO to fast-ripple-HFO [60].

This review informed the age-dependent development of functional abnormalities in S286L-TG, harboring rat S286L-mutant *Chrna4* (corresponding to human S284L-mutant *CHRNA4*). Based on these findings, the proposed age-dependent development of epileptogenesis and ictogenesis of ADSHE in S286L-TG are summarized in Figure 2.

In the initial stage of development of epileptogenesis, loss-of-function S286L-mutant α4β2-nAChRs contribute mainly to two functional abnormalities—enhancement of Erk signaling and excitatory transmission induced by GABAergic disinhibition—in the regions where α4β2-nAChRs are highly expressed [36,53,58,60]. These functional abnormalities have particularly pronounced effects on intra-thalamic tripartite synaptic transmission [55,56,58,61,62,63]. In particular, physiological ripple-HFO during SWS further enhances Erk signaling frequency-dependently, resulting in the gentle increase in trafficking of pannexin1, connexin 43, and P2X7R to the plasma membrane [57,59,60,61,62,63]. Activation of increased hemichannels by physiological ripple-HFO generates persistently increasing releases of L-glutamate, D-serine, ATP, and K^+^ through activated hemichannels due to hemichannel activation persisting over several hours, resulting in further increased Erk signaling and trafficking of hemichannels [60,61,62,63]. These complicated interactions among GABAergic disinhibition, increasing Erk signaling, and hemichannels synergistically enhance each other, generating interictal discharge and/or epileptogenic fast-ripple-HFO [36,60]. Although the combination of intra-thalamic GABAergic disinhibition and physiological ripple-HFO alone cannot generate ADSHE seizures, fast-ripple-HFO is involved in the generation of ADSHE seizures as a major ictogenesis [36,60]. In response to these ADSHE seizures, ripple-HFO and/or fast-ripple-HFO during SWS affects ictogenesis through hemichannel hyperactivation, whereas activation of hemichannels persists over several hours, resulting in the possible generation of multiple or clustering of ADSHE seizures in the same night [36,60,61,62,63].

Functional analyses of S286L-TG have indicated several candidate medications for carbamazepine-resistant epileptic seizures. To date, several clinical studies have already reported that benzodiazepines have favorable effects for the control of ADSHE seizures in patients with the S284L-mutation [1,9,10,14,67]. Indeed, carbamazepine-resistant epileptic seizures—one of the typical clinical features of ADSHE with the S284L-mutation—are plausibly explained by the age-dependent and event-related functional abnormalities in S286L-TG that are composed of GABAergic disinhibition and hyperactivation of tripartite synaptic transmission, which cannot be regulated by voltage-dependent Na^+^ channel inhibition. Furthermore, activated purinergic transmission via increasing astroglial ATP release through enhanced pannexin1 and P2X7R in S286L-TG can be further enhanced by carbamazepine—an established voltage-dependent Na^+^ channel inhibitor—as it presents adenosine A1 receptor antagonist and adenosine A2 receptor agonist activities in both neurons and astrocytes [169,170]. In addition to benzodiazepines, inhibitors of hemichannels, including connexin43, pannexin1, and P2X7R, are also promising therapeutic candidates [171,172].

Based on the GABAergic disinhibition in S284L-TG, chronic administration of furosemide (NKCC1 inhibitor) to S284L-TG before and after the onset of ADSHE has been attempted. Chronic administration of furosemide (over 4 weeks) could not suppress ADSHE seizure frequency after the onset of ADSHE, whereas it prevented the onset of ADSHE by 67% at 4–8 weeks of age (i.e., before the onset of ADSHE) [54]. Therefore, neurotransmission-targeted preventive medication may benefit two-thirds of ADSHE patients with the S284L-mutation, but the results are not clinically satisfactory (complete suppression was not possible). Both in vivo and in vitro functional analyses have demonstrated that connexin43 inhibitors (GAP19 and carbenoxolone), pannexin1 inhibitors (10PANX and probenecid), and P2X7R inhibitor (JNJ47965567) attenuate the enhanced excitatory tripartite synaptic transmission in S286L-TG [36,58,60,61,62,63]. This targeting has the potential to prevent ADSHE onset or suppress seizures before or after onset of ADSHE seizures. In other words, modulating tripartite synaptic transmission can potentially serve as an anti-epileptogenesis therapy.

Effects of systemic administration of the above-described agents on the seizure frequency of S286L-TG have not been determined, but their inhibitory effect on the hyperactivated tripartite synaptic transmission within the epileptogenic region and networks/circuits in S286L-TG has been reported. Both Gap19 and 10PANX are selective mimetic peptides inhibitors derived from connexin43 and pannexin1, respectively. When conjugated with TAT sequence (TAT-Gap19), it gains cell-penetrating properties and has been shown to permeate the blood–brain barrier (BBB) in vivo; however, direct evidence regarding BBB permeability of 10PANX is limited [173,174,175].

Carbenoxolone is a candidate agent for comprehensive inhibition of astroglial transmission, including connexin43, pannexin1, and P2X7R [60,61,62,171]. Local administration of carbenoxolone (100 µM) into the PPN using the reverse dialysis method not only decreased basal L-glutamate and ACh levels in MoTN, STN, and RTN during wakefulness and SWS but also suppressed increasing releases of ACh and L-glutamate during ADSHE seizure in S286L-TG [63]. Local administration of carbenoxolone (100 µM) but not carbamazepine into the OFC or MDTN decreased basal and AMPA-evoked releases of ATP and L-glutamate in S286L-TG [58]. Local administration of carbenoxolone (100 µM) into the OFC also suppressed the persistently increasing release of ATP and L-glutamate induced by P2X7R agonist, BzATP, in S286L-TG [62]. However, various off-target effects of carbenoxolone, such as inhibition of voltage-gated Ca^2+^ channels, 11β-hydroxysteroid dehydrogenase, GABA, and AMPA receptor-mediated synaptic transmission, indicate that all largely affect excitability and complicate the interpretation of pharmacological studies with this compound [171].

Probenecid is known as a relatively selective inhibitor of pannexin1-hemichannel (IC_50_: 150 μM) and P2X7R (IC_50_: 200 µM) compared to carbenoxolone [61,163,176,177]. Local administration of probenecid (300 µM) into the PPN using the reverse dialysis method decreased basal L-glutamate and ACh levels in their projection regions, such as MoTN, STN, and RTN, during SWS and ADSHE seizure but not wakefulness [62,63]. Probenecid has been used to prevent gout attacks for several decades via inhibition of the family of organic anion transporters (IC_50_: 5–15 μM). Considering the standard therapeutic dose of probenecid (1–2 g/day) for gout, targeting pannexin1-hemichannels may necessitate supratherapeutic concentrations, raising concerns regarding clinical feasibility and safety.

JNJ47965567 is developed as a selective P2X7R antagonist [178]. In the OFC, larger releases of L-glutamate and ATP induced by local administration of BzATP (P2X7R agonist: 1–100 μM) in S286L-TG compared to wild-type using the reverse dialysis method have been demonstrated [62]. Local perfusion of 1 μM JNJ47965567 into the OFC inhibited BzATP-evoked releases of ATP and L-glutamate [62]. Recently, an in vitro study reported that JNJ47965567 suppressed Ca^2+^ influx in human iPSC-derived neurons, suggesting its potential antiepileptic effects [179]; however, further continuous studies remain essential for the development of a novel antiseizure medication.

## 7. Remaining Challenges

S286L-TG has already revealed several findings that are not apparent in single-cell models, including that S284L-mutant α4β2-nAChRs contribute to the development of ADSHE epileptogenesis/ictogenesis by affecting various transmission abnormalities, including tripartite synaptic transmission in ADSHE circuits [36]. However, further efforts are needed to establish the validity and reliability of rodent epilepsy models. Rat models have demonstrated more robust face validity compared to mouse models [20,25,45,46,47,49,52,53]. Indeed, discrepancies in the face validity between transgenic rat and knock-in mouse models suggest that the genetic backgrounds, general structures, and dimensions of the relevant brain regions of rats are more appropriate for the development of ADSHE pathogenesis than those of mice [36]. Therefore, verification of the importance of the different candidate backgrounds in further genetic rodent models using current techniques for the development of genetic animal models, such as editing, is expected to contribute to further progress in elucidating the pathogenesis of ADSHE.

In S286L-TG, functional abnormalities in astroglial transmission induced by loss-of-function S284L-mutant α4β2-nAChRs play an important role in the development of epileptogenesis/ictogenesis; meanwhile, in other mutations, the impacts of gain-of-function V287L-mutant α4β2-nAChRs on tripartite synaptic transmission remain to be clarified. Exploring the functional abnormalities in V287L-mutant ADSHE models can provide further understanding regarding the pathomechanisms of ADSHE. Additionally, valid ADSHE models harboring ADSHE-mutant *DEPDC5* remain to be established. *DEPDC5* and the GATOR1 complex inhibit downstream mTOR signaling, which is required for neural stem cell differentiation, neuroprogenitor cell proliferation, dendritic formation, synaptic transmission and plasticity, and the development of neural network activity [180,181]. Considering that the majority of patients with *DEPDC5* mutations have structural brain abnormalities, and even though ADSHE caused by *DEPDC5* and *CHRNA4* mutations display similar symptomatology, it may be necessary to consider them as having different pathomechanisms. However, upregulation of Erk signaling activates mTOR signaling via suppression of *TSC* [181]. This hypothesis must be confirmed using established, valid ADSHE models harboring the *DEPDC5* mutant.

## 8. Conclusions

This review introduced candidate pathomechanisms of ADSHE based on functional analyses using ADSHE model rats, S284L-TG and S286L-TG. Surprisingly, the pathomechanisms currently identified differed markedly from those initially anticipated when gene mutation was discovered in ADSHE pedigrees three decades ago, although the loss-of-function S284L-mutant nAChRs (rat S286L-mutant nAChRs in S286L-TG and S284L-TG) generate impaired GABAergic transmission as the initial stage of epileptogenesis. Subsequently, the combination of pathological GABAergic disinhibition with propagation of physiological ripple-HFO enhances Erk signaling and astroglial hemichannel function during neurodevelopment as the main epileptogenesis of ADSHE. Following the acquisition of epileptogenesis, physiological ripple-HFO during SWS can generate epileptogenic fast-ripple-HFO, which played a critical role in seizure initiation (ictogenesis). These findings suggest that agents capable of suppressing tripartite synaptic transmission may represent promising therapeutic candidates for ADSHE resistant to conventional antiseizure medication.

## Figures and Tables

**Table 1:** Changes in transmitter levels during the transition from wakefulness to SWS in S286L-TG after the ADSHE seizure onset.

	Ach		GABA		L-Glutamate	
	Wild	S286L-TG	Wild	S286L-TG	Wild	S286L-TG
PPN	↓	↓	→	→	↓	→
RTN	↓	↓	↓	→	↓	→
MoTN/MDTN	↓	↓	↓	→	↓	→
M2C/OFC	↓	↓	→	→	↓	→
STN	↓	↓	→	→	↓	→
SNr					↓	→

SWS: slow-wave sleep, wild: wild-type rats, PPN: pedunculopontine tegmental nucleus, RTN: reticular thalamic nucleus, MoTN: motor thalamic nuclei, MDTN: mediodorsal thalamic nucleus, M2C: secondary motor cortex, OFC: orbitofrontal cortex, STN: subthalamic nucleus, SNr: substantia nigra pars reticulata. ↓: significantly (*p* < 0.05, N = 6) decreasing extracellular levels with the transition from wakefulness to SWS of S286L-TG (8–10 weeks of age) using microdialysis study. →: unchanging extracellular levels with the transition from wakefulness to SWS.

**Table 2:** Extracellular transmitter levels of S286L-TG compared to wild-type.

	ACh		GABA		L-Glutamate	
	Wakefulness	SWS	Wakefulness	SWS	Wakefulness	SWS
PPN	→	→	→	→	↑	↑
RTN	→	→	→	→	↑	↑
MoTN/MDTN	→	→	→	→	↑	↑
M2C/OFC	→	→	→	→	↑	↑
STN	→	→	→	→	↑	↑
SNr					↑	↑

↑: significantly (*p* < 0.05, N = 6) larger levels of S286L-TG than wild-type using microdialysis study.→: equivalent between S286L-TG and wild-type (8–10 weeks of age).

## Data Availability

The raw data supporting the conclusions of this article will be made available by the authors upon request.

## Abbreviations

The following abbreviations are used in this manuscript:

ACh	acetylcholine
nAChRs	nicotinic acetylcholine receptors
ADSHE	autosomal-dominant sleep-related hypermotor epilepsy
DC	direct-current
ENW	episodic nocturnal wandering
HFO	high-frequency oscillation
M2C	secondary motor cortex
MDTN	mediodorsal thalamic nucleus
MoTN	motor thalamic nuclei
NPA	nocturnal paroxysmal arousal
NPD	nocturnal paroxysmal dystonia
non-REM	non-rapid eye movement
OFC	orbitofrontal cortex
PPN	pedunculopontine nucleus
RTN	reticular thalamic nucleus
SHE	sleep-related hypermotor epilepsy
SNr	substantia nigra pars reticulata
STN	subthalamic nucleus
SWS	slow-wave sleep
